# Ökologische Nachhaltigkeit in der Urologie

**DOI:** 10.1007/s00120-025-02720-4

**Published:** 2025-10-30

**Authors:** Bernd Wullich, Holger Borchers, Christian Eggersmann, Maximilian Burger, Marlene Thöne

**Affiliations:** Urologische und Kinderurologische Klinik, Universitätsklinikum Erlangen, Erlangen, Deutschland

**Keywords:** Klimaschutz, Klimaresilienz, Klimaneutralität, Umweltschutz, Ressourcenschonung, Climate protection, Climate resilience, Climate neutrality, Environmental protection, Resource conservation

## Abstract

Das Thema ökologische Nachhaltigkeit hat in den letzten Jahren nicht nur im gesellschaftlichen Diskurs, sondern auch im Gesundheitswesen enorm an Bedeutung gewonnen. Der Gesundheitssektor in Deutschland trägt mit > 5 % erheblich zu den gesamten CO_2_-Emissionen bei und verbraucht etwa ebenso viele der verarbeiteten Rohstoffe. Auch für die Urologie besonders relevante Bereiche wie Arzneimittel, Medizinprodukte und Instrumentenlogistik zählen neben baulichen Maßnahmen, dem Betrieb von Kliniken und Praxen sowie der Lebensmittelversorgung zu den intensivsten Ressourcenverbrauchern. Vor diesem Hintergrund hat die Deutsche Gesellschaft für Urologie e. V. (DGU) die Dringlichkeit erkannt und eine Nachhaltigkeitsstrategie entwickelt, die alle Sektoren des Faches einbezieht und konkrete Perspektiven für einen verantwortungsvollen Umgang mit den bestehenden Ressourcen liefern soll.

## Abstract (en)

Environmental sustainability has become increasingly important in recent years, not only in social discourse but also in the healthcare sector. The healthcare sector in Germany contributes significantly to total CO_2_ emissions, accounting for over 5%, and consumes roughly the same amount of resources. Areas that are particularly relevant to urology, such as pharmaceuticals, medical devices, and instrument logistics, are among the most intensive consumers of resources, alongside construction measures, the operation of clinics and practices, and food supply. In light of this, the German Society of Urology (DGU) has recognized the urgency of the situation and developed a sustainability strategy that includes all sectors of the field and aims to provide concrete perspectives for the responsible use of existing resources.

## Einleitung

Das vorliegende Papier greift den Status quo bezüglich ökologischer Nachhaltigkeit in der Urologie auf und beschreibt notwendige nächste Schritte. Es wurde an internationale Fachpositionierungen angelehnt und auf Basis der aktuellen Evidenzlage erstellt. Ziel ist es, Urolog*innen zu motivieren und zu unterstützen, ökologische Nachhaltigkeit in ihren Arbeitsalltag zu integrieren. Der Anspruch bleibt unverändert: die bestmögliche Versorgung unserer Patient*innen. Doch wenn Umweltveränderungen und der Klimawandel künftig die Sicherheit unserer Patient*innen selbst gefährden, müssen wir handeln. Urolog*innen können im interdisziplinären Zusammenschluss mit Anästhesie, Pflege, Technik, Verwaltung und Industrie eine Vorreiterrolle einnehmen. Ein klimaneutrales Gesundheitswesen bis 2045 ist ohne Beteiligung aller Fachdisziplinen nicht realistisch. In anderen Teilen der Welt (z. B. Großbritannien, Skandinavien) wird bereits gezielt daran gearbeitet.

Diese Veränderungen müssen „*bottom up*“ durch Fachabteilungen und „*top down*“ durch Management, Politik und Industrie unterstützt werden.

Mit diesem Positionspapier gibt die Deutsche Gesellschaft für Urologie e. V. (DGU) ein klares Signal: Wir sind bereit, uns unserer Verantwortung zu stellen – im Sinne von Sicherheit unserer Patient*innen, Versorgungsqualität und *Planetary Health*. Eine DGU-Mitgliederbefragung Anfang 2025 zeigte eine grundsätzliche Bereitschaft der Urolog*innen, sich aktiv an einer nachhaltigen Gestaltung des Fachs zu beteiligen.

## Auswirkungen der operativen Urologie

Die operative Urologie ist ressourcenintensiv. Roboter-assistierte Verfahren haben oft einen höheren ökologischen Fußabdruck als offene oder laparoskopische Eingriffe [2]. Dabei spielen Faktoren wie Energiebedarf, CO_2_-Erzeugung, Materialeinsatz und Aufbereitungsaufwand eine Rolle. Dennoch bleibt die oberste Priorität die Sicherheit unserer Patient*innen und der medizinische Outcome. Ein bewusster Umgang mit Operationen (z. B. die Vermeidung unnötiger Eingriffe, eine volumen- und erfahrungsbasierte Bündelung von Eingriffen), eine ressourcenschonende Planung sowie die interdisziplinäre Zusammenarbeit – insbesondere mit der Anästhesiologie – können zur Reduktion der Emissionen beitragen. Der gezielte Verzicht auf besonders klimaschädliche Anästhetika wie Sevofluran ist vielerorts bereits erfolgreich etabliert.

## Verwendung von Einwegartikeln

Die Frage nach dem Verhältnis zwischen Einweg- und Mehrwegprodukten ist eine der zentralen Herausforderungen ökologisch nachhaltiger OP- und Stationsorganisation. Während Einwegartikel aus hygienischer und logistischer Sicht Vorteile bieten, gehen sie häufig mit einem deutlich höheren Material- und Energieverbrauch einher.

Mehrwegprodukte weisen – trotz des Energieaufwands für Reinigung und Sterilisation – in den meisten Fällen eine günstigere Ökobilanz auf als ihre Einweg-Pendants [3, 4]. Lebenszyklusanalysen (LCA) zeigen, dass etwa chirurgische Instrumente oder OP-Mäntel im Mehrwegformat emissionsärmer sind, sofern die Aufbereitung effizient erfolgt und mit Strom aus möglichst regenerativen Quellen betrieben wird.

Einzelne Studien weisen darauf hin, dass „Single-use-Artikel“ unter bestimmten Rahmenbedingungen ökologisch gleichwertig oder vorteilhafter sein können – etwa bei besonders komplexen Eingriffen oder unter Einsatz kohlebasierten Strommixes (z. B. Australien). Diese Ergebnisse lassen sich jedoch nicht ohne Weiteres auf den deutschen Kontext übertragen, der einen zunehmend dekarbonisierten Energiemix aufweist.

Bei der Interpretation von Ökobilanzen ist zudem besondere Vorsicht geboten: Viele Studien werden von Herstellerfirmen selbst in Auftrag gegeben oder berücksichtigen nicht alle Lebenszyklusphasen gleichgewichtig (z. B. Produktion, Transport, Nutzung, Entsorgung). Auch die Anzahl der Wiederverwendungen, die in den Ökobilanzstudien berücksichtigt wurde, erschweren ihre korrekte Einordnung. Solche Limitationen müssen bei der Entscheidungsfindung berücksichtigt und die jeweiligen Bedingungen (Fallzahlen, Aufbereitungspfade, Transportwege und das Entsorgungsmanagement) beachtet werden. Nicht in allen LCAs sind Sensitivitätsanalysen enthalten, die diese Elemente genauer prüfen. Eine kürzlich erschienene Studie betrachtet in der aktuellen Literatur befindliche Analysen zu Nachhaltigkeitsaspekten von Einwegendoskopen gegenüber Mehrwegendoskopen und identifiziert einerseits Hinweise auf die zusätzliche Umweltbelastung durch die Einweginstrumente, andererseits aber auch einen Bedarf an weiteren Studien in diesem Feld [5].

## Entsorgungsmanagement

Täglich entstehen pro Krankenhausbett ca. 6 kg Abfall [6]. Vieles davon wäre vermeidbar oder recycelbar. Doch häufig werden ganze Sets geöffnet, ohne dass alle Bestandteile benötigt werden. Auch das Tragen unsteriler Handschuhe bei Tätigkeiten ohne Infektionsrisiko, das unnötige Ausdrucken digital vorliegender Informationen und eine nicht vorhandene Recyclingstruktur sind häufige Probleme.

Nachhaltiges Abfallmanagement sollte daher ein integraler Bestandteil des Klinikbetriebs sein. Dabei kann das sog. 5R-Konzept, abgeleitet aus der Abfallhierarchie der Europäischen Union 2008/98/EG (Waste Framework Directive), als strukturierende Leitlinie angewandt werden:

- „refuse“ – unnötige Produkte oder Prozesse ablehnen,
- „reduce“ – Verbrauch reduzieren,
- „reuse“ – wiederverwendbare Produkte einsetzen,
- „recycle“ – verwertbare Materialien getrennt entsorgen,
- „rethink“ – bestehende Abläufe überdenken.

Insbesondere OP-nahe Bereiche und Polikliniken bieten ein hohes Potenzial zur aktiven Abfallvermeidung, ohne die Patient*innensicherheit zu beeinträchtigen: In der Vorbereitung geplanter Eingriffe z. B. könnten Abfalltrennung und Recyclingansätze umgesetzt werden. Ein flächendeckendes Monitoring oder Analysen von Fehlwürfen könnten das Bewusstsein im Team stärken, regelmäßige Schulungen die Aufmerksamkeit auf das Thema lenken.

## Mobilität

Ein erheblicher Anteil der klinikbezogenen Emissionen entfällt auf den Verkehr: Anfahrten von Personal und Patient*innen sowie Materialtransporte. Förderprogramme wie Jobtickets, Bike-Leasing und Homeoffice-Optionen für administrative Tätigkeiten und Telemedizin werden vereinzelt genutzt, bieten aber noch erhebliches Einsparpotenzial. Auch Fortbildungsveranstaltungen können durch hybride Formate Emissionen senken – ohne die wissenschaftliche Qualität zu beeinträchtigen.

## Telemedizin

Gerade in der Urologie eignen sich viele Termine zur telemedizinischen Durchführung – etwa Befundbesprechungen oder präoperative Gespräche. Eine aktuelle Studie zeigt, dass telemedizinische Prämedikation von Patient*innen gut angenommen wird und logistisch sowie ökologisch sinnvoll ist [7]. Durch digitale Formate können bei gleichbleibender medizinischer Qualität Fahrtwege, Wartezeiten und Ressourcen eingespart werden, was insbesondere auch mobilitätseingeschränkten und in ländlichen Gebieten lebende Patient*innen zugutekäme [8].

## Bau und Energie

Ein erheblicher Teil der Emissionen im Gesundheitswesen entsteht durch den Bau und Betrieb von Gebäuden. Energetische Sanierungen, Photovoltaik, LED-Beleuchtung und Wärmedämmung können den CO_2_-Ausstoß drastisch senken. Neubauten sollten nach modernsten Nachhaltigkeitsstandards und widerstandsfähig gegenüber Naturkatastrophen wie Überschwemmungen, extremer Hitze und Wasserknappheit, Feinstaub- und Rauchbelastungen geplant werden [9], auch wenn initial höhere Investitionen nötig sind. Diese amortisieren sich langfristig durch geringere Betriebskosten und bessere CO_2_-Bilanzen. Auch Renovierungen von Bestandsgebäuden sollten entsprechend der notwendigen Klima- und Umweltresilienz vorgenommen werden, insbesondere in kritischen Bereichen wie zentralen Notaufnahmen, Intensivstationen und dem OP. Solche Maßnahmen wirken außerdem präventiv z. B. Stromausfällen entgegen und dienen letztendlich der Versorgungssicherheit.

## Forschung und Lehre

Nachhaltigkeit spielt in der medizinischen Forschung bislang eine untergeordnete Rolle. Auch existiert bisher keine einheitliche Struktur zur Messung von CO_2_-Emissionen, z. B. durch Standardisieren von Maßeinheiten. Lebenszyklusanalysen medizinischer Produkte, planetare Auswirkungen neuer Therapien, ressourcenschonende Studienkonzepte aber auch vertiefende Untersuchungen des Klimawandels als Risikofaktor uro(onko)logischer Erkrankungen sollten künftig vermehrt Eingang in die urologische Forschung finden [10]. Annahmen wie der Zeithorizont des „global warming potentials“ (GWP), definierte Systemgrenzen und z. B. die einheitliche Benutzung der funktionellen Einheit „kg CO_2_-Äquivalente pro Eingriff“ sollten etabliert werden. Auch die medizinische Ausbildung muss ökologisches Grundwissen stärker vermitteln – hierzu gibt es erste curriculare Initiativen. Eine Vereinheitlichung von Methoden und Messgrößen würde hier zusätzlichen Anreiz verschaffen, vermehrt reproduzierbare Studien zum Transfer in Praxis und Lehre durchzuführen.

## Ko-Benefits

Viele Nachhaltigkeitsmaßnahmen bieten nicht nur ökologische, sondern auch gesundheitliche und ökonomische Vorteile. Die EAT-Lancet-Kommission (2019) beschreibt z. B. eine Ernährung, die sowohl Umwelt als auch Gesundheit schützt [11]. Ebenso verbessern sich durch aktive Mobilität (Radfahren, Gehen) kardiovaskuläre Probleme und psychisches Wohlbefinden. Telemedizin kann durch Verringerung von Wegekosten und -zeit gerade für Patient*innen mit langem Anfahrtsweg oder körperlichen Einschränkungen deutliche Erleichterung verschaffen. Insgesamt führt ein nachhaltiges Wirtschaften in allen Bereichen der Gesundheitsversorgung zu einer langfristigen Versorgungsgerechtigkeit und -sicherheit. Dies gewährt neben den direkten (planetar) gesundheitlichen Benefits auch eine indirekte, generationenübergreifend faire und inklusive Versorgung. Solche Ko-Benefits sollten stärker kommuniziert und genutzt werden.

## Ausblick

Mit diesem Positionspapier stellt sich die DGU ausdrücklich in die Rolle eines *Option Leaders* – nicht nur als Fachgesellschaft, sondern auch als Gestalterin einer nachhaltigen Zukunft für die Urologie. Die Roadmap, die auf dem Jahreskongress 2025 in Hamburg präsentiert wurde, definiert zentrale Schritte: durch Wissenstransfer und Sensibilisierung, systematische Forschungsarbeiten und konkrete Pilotprojekte soll eine nachhaltige Perspektive und fundierte Evidenz geschaffen werden. In Zusammenarbeit mit anderen Fachbereichen, Politik, Verwaltung und Industrie soll diese Transformation umgesetzt werden. Ziel ist eine klimaresiliente, verantwortungsbewusste Urologie im Sinne des „sustainable development goals“ (SDG) 3 „*Health and Well being*“.
